# Supplementary material for: Bioadhesive interface for marine sensors on diverse soft fragile species
Source: Nat Commun. 2024 Apr 16;15:2958. doi: 10.1038/s41467-024-46833-4 (PMC11021473; doi:10.1038/s41467-024-46833-4)
Supplement: Supplementary file 1 — Supplementary Information [file 41467_2024_46833_MOESM1_ESM.pdf]

**Supplementary Information**

# Bioadhesive Interface for Marine Sensors on Diverse Soft Fragile Species

Camilo Duque Londono[1,†], Seth F. Cones[3,†], Jue Deng[1,6†], Jingjing Wu[1], Hyunwoo Yuk[1,5], David E. Guza[7], T. Aran Mooney[4,*], Xuanhe Zhao[1,2,*]

[1] Department of Mechanical Engineering, Massachusetts Institute of Technology, Cambridge, MA, USA.

[2] Department of Civil and Environmental Engineering, Massachusetts Institute of Technology, Cambridge, MA, USA.

[3]Massachusetts Institute of Technology and Woods Hole Oceanographic Institution Joint Program in Oceanography/Applied Ocean Science & Engineering, Cambridge, MA 02139, USA

[4] Biology Department, Woods Hole Oceanographic Institution, Woods Hole, MA 02543, USA

[5] Present address: SanaHeal, Inc., Cambridge, MA, USA

[6] Present address: Academy for Engineering and Technology, Fudan University, Shanghai 200433, China

[7] Present address: Applied Engineering Solutions LLC., Columbus, OH, USA

[†] These authors contributed equally

*Corresponding authors: zhaox@mit.edu, amooney@whoi.edu

**This PDF includes the following:**

Supplementary Figures 1 to 8

**Other Supplementary Materials for this manuscript include:**

Supplementary Movies 1 to 4

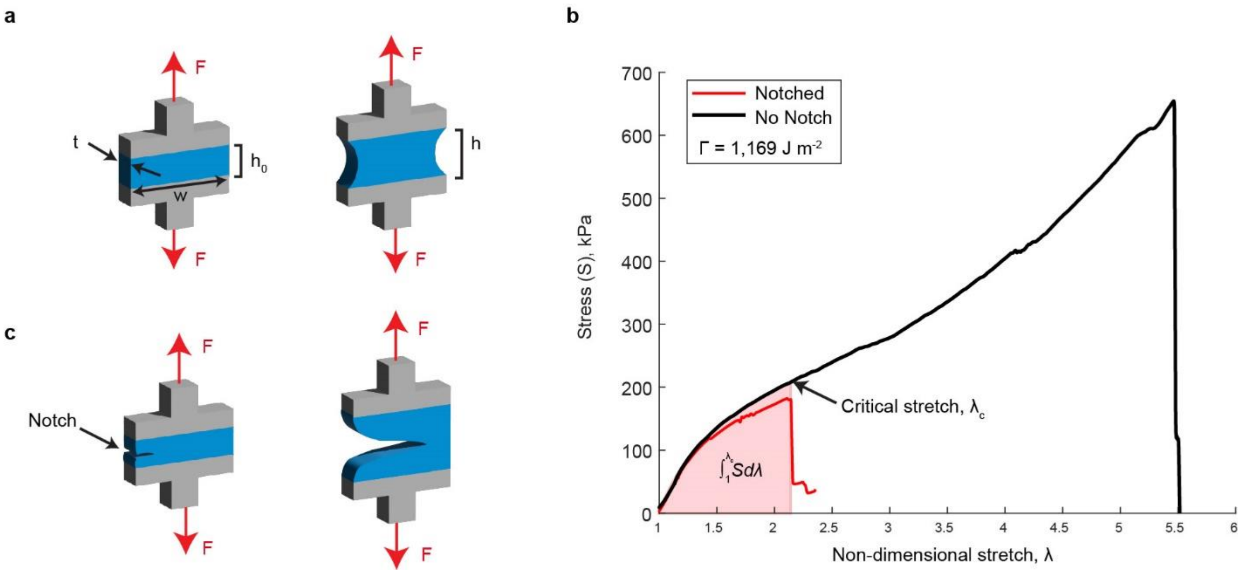


**Supplementary Fig. 1 | Fracture toughness of hydrogel adhesive. a & c,** Schematic of the pure-shear tensile test setup. A sample with no notch (a) is stretched to failure to create a measured nominal stress vs. stretch curve. A sharp crack is introduced into a fresh sample (c) with identical dimensions and stretched to failure. **b,** Nominal stress vs. stretch curve for the notched and unnotched samples. The shaded area is the mechanical work applied up until the critical stretch, which is used to calculate fracture toughness.

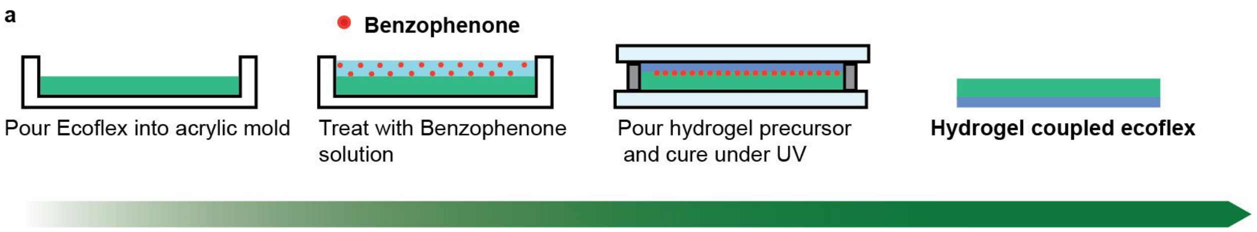


**Supplementary Fig. 2 | Coupling of hydrogel adhesive to Ecoflex. a,** Fabrication of hydrogel coupled Ecoflex layer of the BIMS. Cured Ecoflex is treated with a benzophenone solution to prepare the surface for hydrogel coupling. Then, the hydrogel precursor can be poured directly onto the treated surface and cured in a UV chamber for 30 mins.

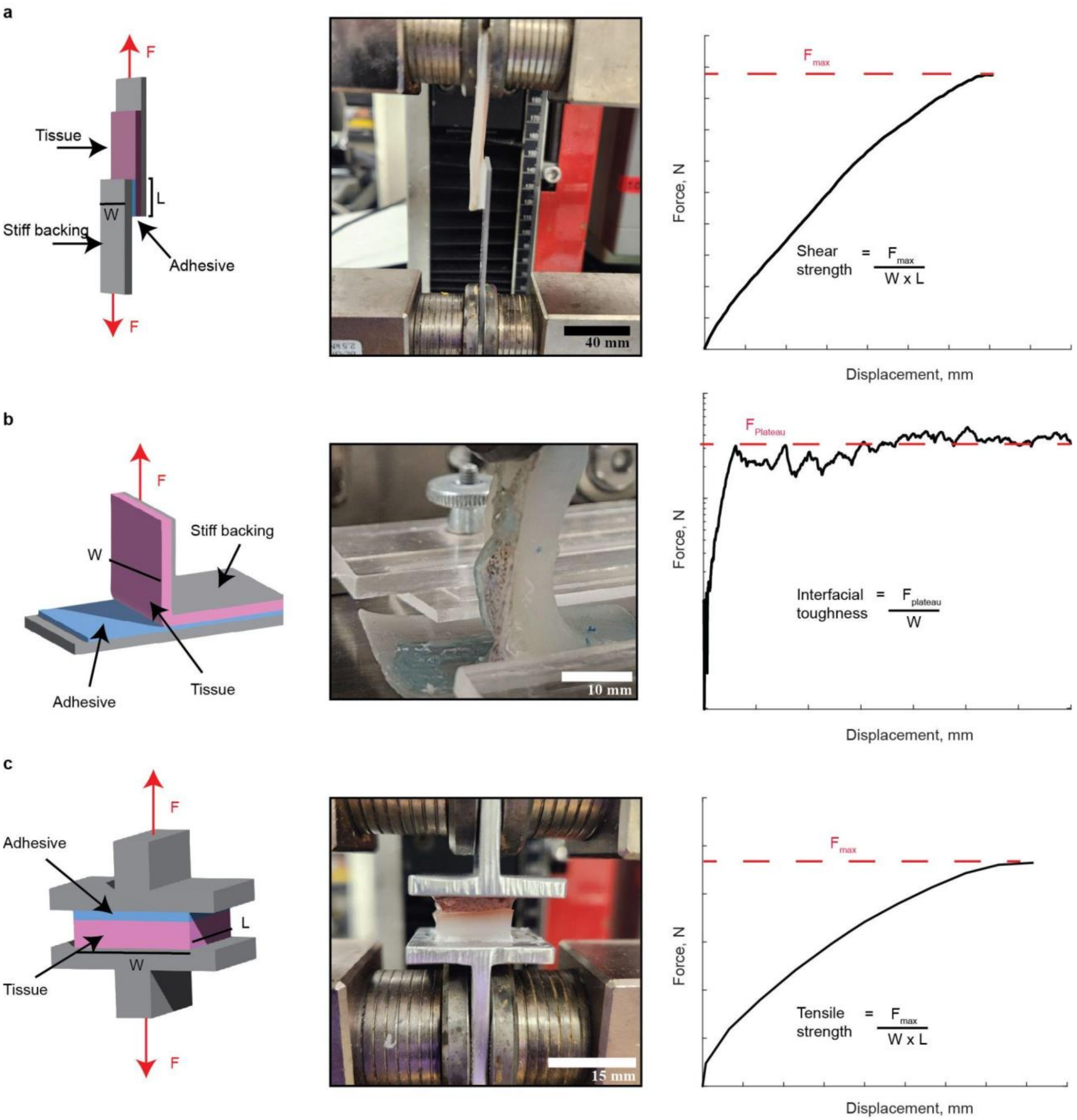


**Supplementary Fig. 3 | Mechanical tests of adhesion strength on marine tissues. a,** Test setup and force vs. displacement curve of lap shear test used to measure shear strength of the BIMS on marine tissues. **b,** Test setup and force vs. displacement curve of 90-degree peel test of the BIMS on marine tissues to measure interfacial toughness. **c,** Test setup and force vs. displacement curve of standard tensile test to measure tensile strength of the BIMS on marine tissues.

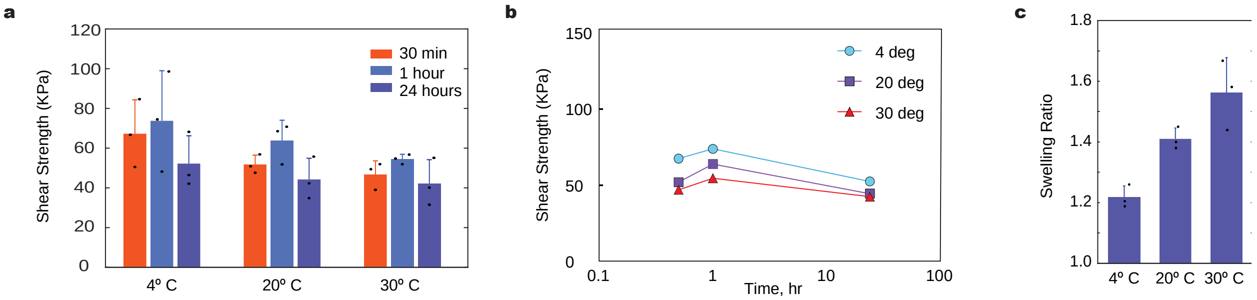


**Supplementary Fig. 4 | Swelling and adhesion stability at different temperatures. a**,**b**, shear strength of the BIMS on squid tissue over 24 hours at three representative temperatures. **c**, Adhesive swelling ratio at three different temperatures. Values in **a**,**b**, and **c** are mean and standard deviation where error bars are present (n=3 independent samples).

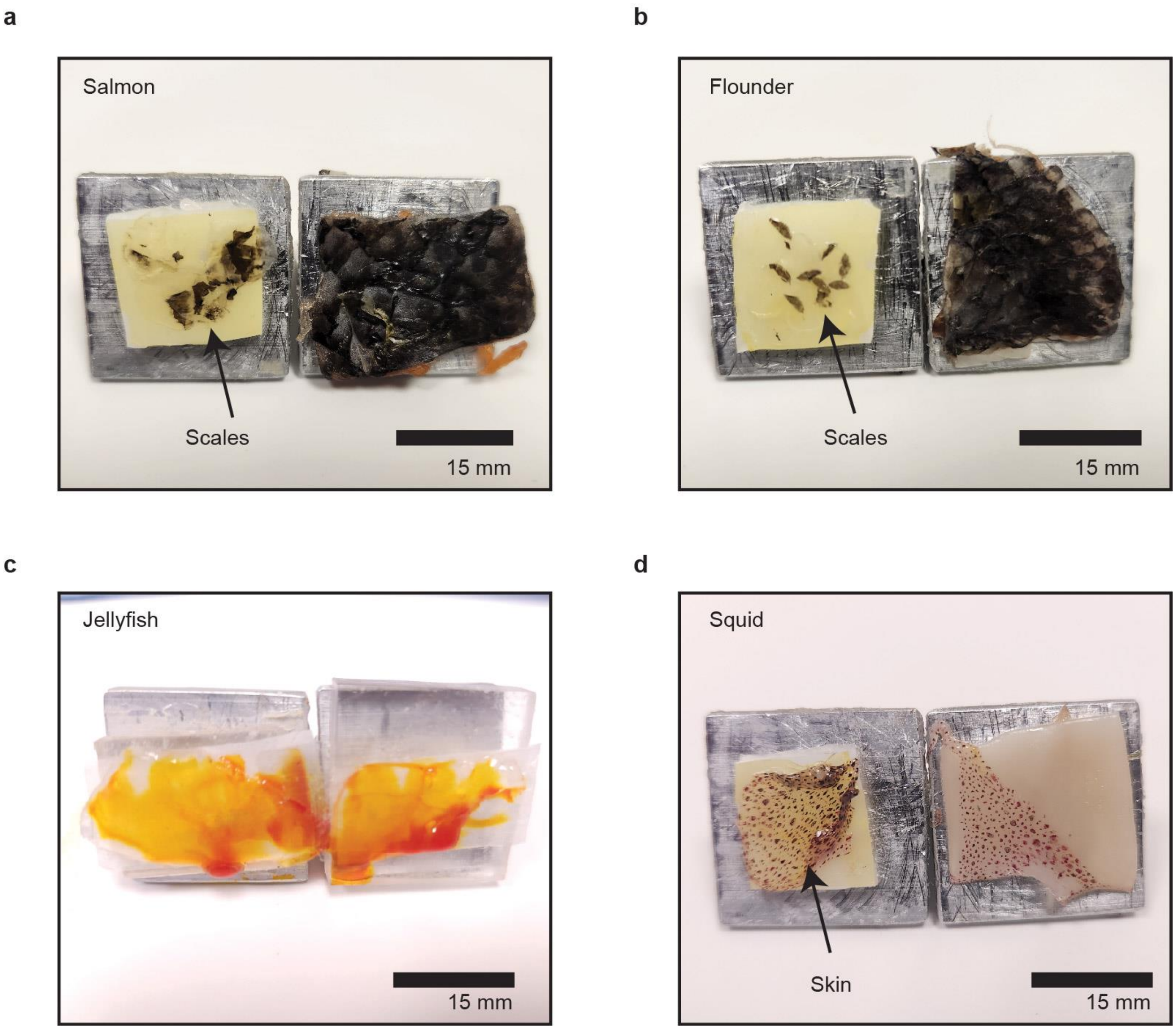


**Supplementary Fig. 5 | Cohesive failure of adherent during tensile testing. a-d,** Images of tensile test results for salmon, flounder, jellyfish, and squid. All tests were conducted within one hour of application, which caused cohesive failure at the skin/scale muscle interface rather than the adhesive failure of the BIMS. In every test, the adhesive remained on the substrate rather than the tissue, indicating a stronger bond on the substrate-adhesive interface. Jellyfish tissue (c) was dyed with red food coloring for visual purposes.

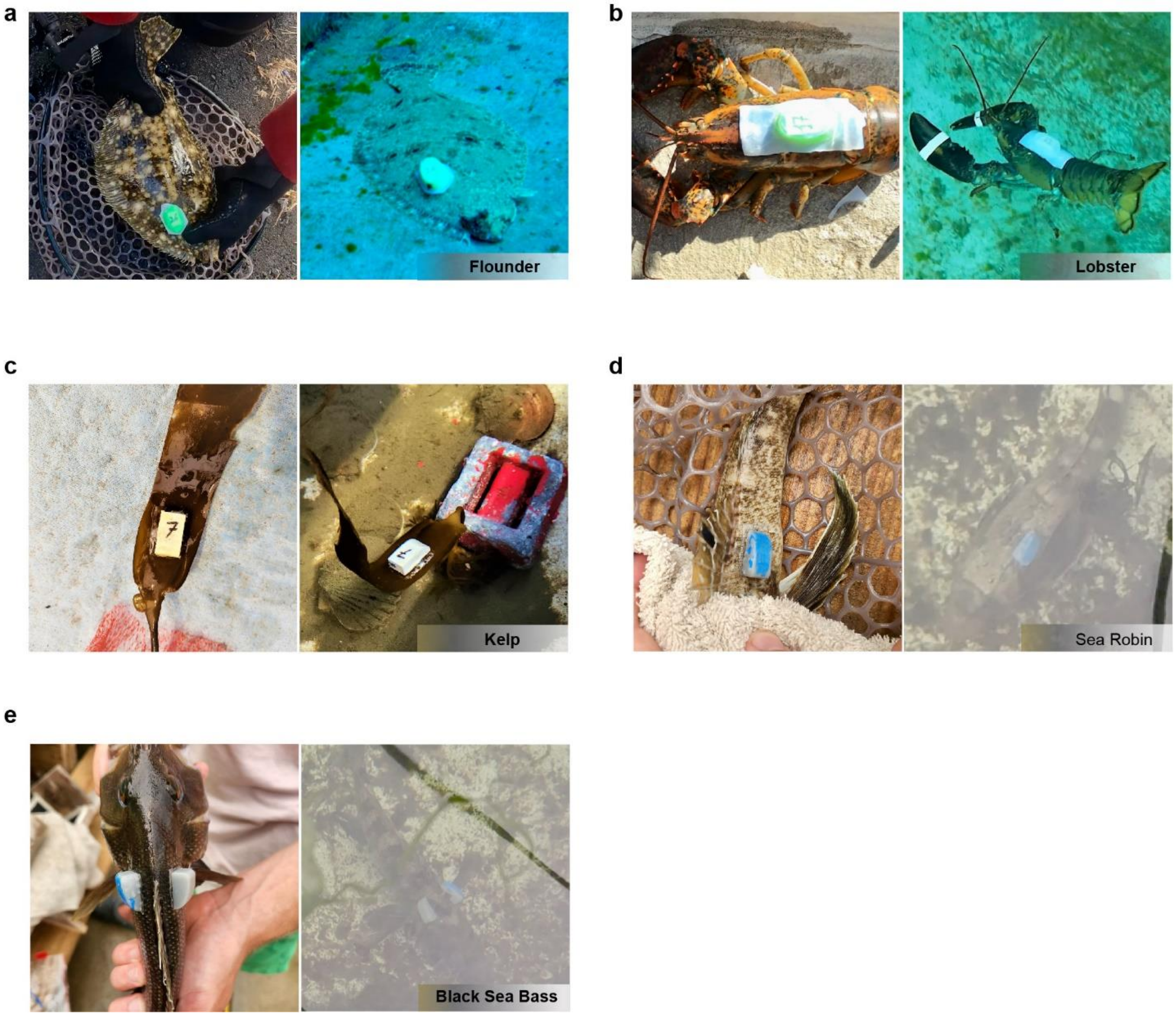


**Supplementary Fig. 6 | Images and adhesion time for various marine species. a-e,** Images of the BIMS applied to flounder, lobster, kelp, sea robin and sea bass before (left) and after (right) returning to the water.

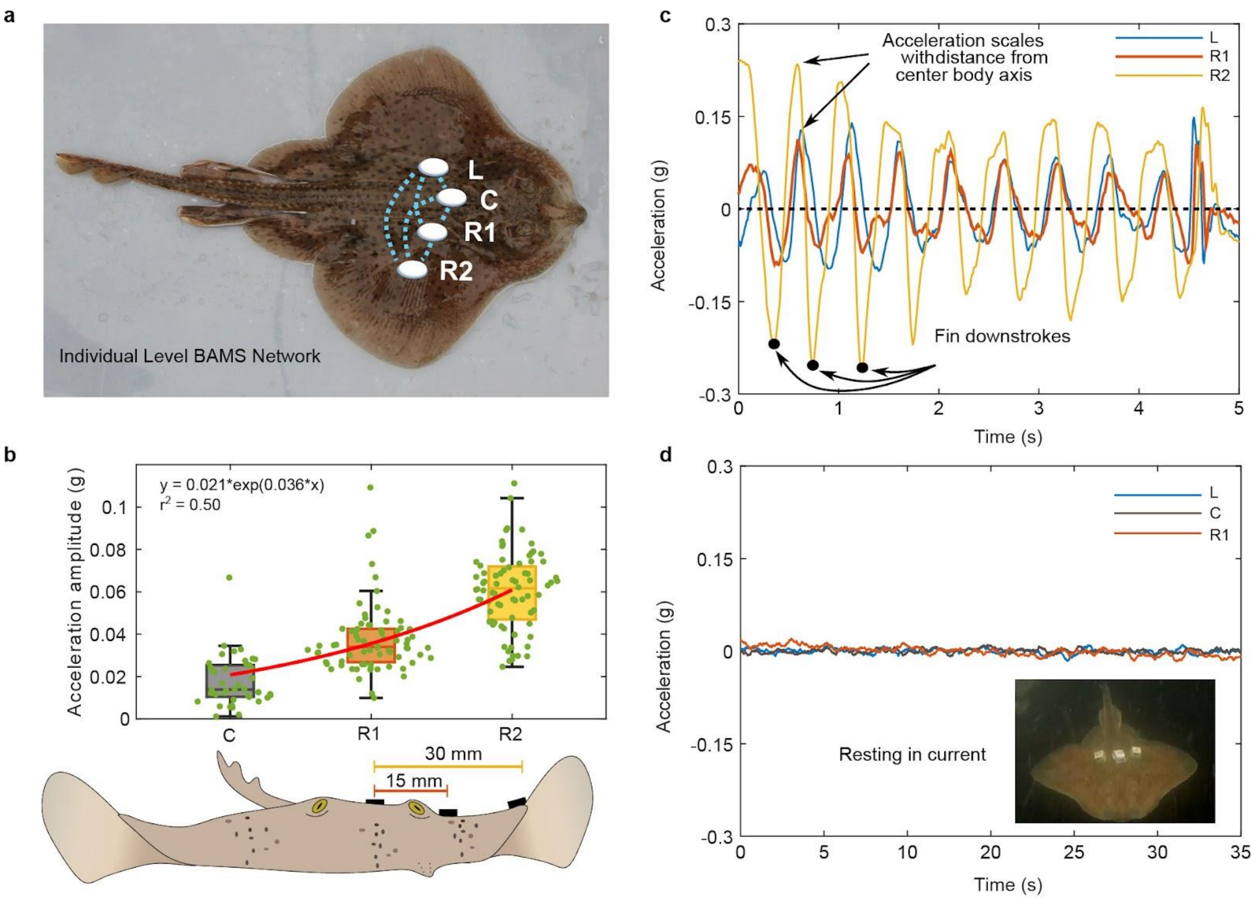


**Supplementary Fig. 7 | Individual-level sensor network for whole-body movements using BIMS**. **a**, Photograph of the little skate (*Leucoraja erinacea*) and the location of four sensors on the left pectoral fin (L), central body axis (C), and right pectoral fins (R1, R2). **b**, Peak acceleration amplitude during 20 cm $s^{-1}$ swimming at three body positions. The BIMS network data were fit into a model to map acceleration across the pectoral fin. The boxplot centerline marks the median value and the upper and lower box edges denote the extent of the interquartile range. The whiskers mark the minimum and maximum non-outlier data, and the green dots depict the raw data. **c**, Acceleration data during active swimming at the left and right pectoral fins. **d**, The BIMS measured resting behaviors, which are vital for this benthic species.

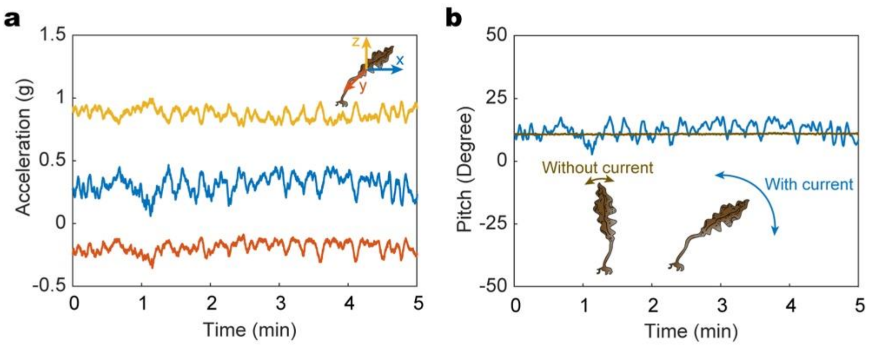


**Supplementary Fig. 8 | Seaweed movement and water current detection via BIMS.** Real-time monitoring of acceleration (a) and pitch (b) of kelp blade via the BIMS, showing the capability of ambient water current detection.
