# Supplementary material for: Bioadhesive interface for marine sensors on diverse soft fragile species
Source: Nat Commun. 2024 Apr 16;15:2958. doi: 10.1038/s41467-024-46833-4 (PMC11021473; doi:10.1038/s41467-024-46833-4)
Supplement: Supplementary file 3 — Description of Additional Supplementary Files [file 41467_2024_46833_MOESM3_ESM.pdf]

Description of Additional Supplementary Files

File Name: Supplementary Movie 1
Description: BIMS application process.

File Name: Supplementary Movie 2
Description: *Squid* jetting while equipped with the BIMS.

File Name: Supplementary Movie 3
Description: Large tank in-vivo testing.

File Name: Supplementary Movie 4
Description: BIMS field experiment.
