# Supplementary material for: Bioadhesive interface for marine sensors on diverse soft fragile species
Source: Nat Commun. 2024 Apr 16;15:2958. doi: 10.1038/s41467-024-46833-4 (PMC11021473; doi:10.1038/s41467-024-46833-4)
Supplement: Supplementary file 8 — Reporting Summary [file 41467_2024_46833_MOESM8_ESM.pdf]

nature portfolio

Corresponding author(s): Xuanhe Zhao, T. Aran Mooney

Last updated by author(s): Jan 24, 2024

Reporting Summary

Nature Portfolio wishes to improve the reproducibility of the work that we publish. This form provides structure for consistency and transparency in reporting. For further information on Nature Portfolio policies, see our Editorial Policies and the Editorial Policy Checklist.

Statistics

For all statistical analyses, confirm that the following items are present in the figure legend, table legend, main text, or Methods section.

n/a	Confirmed	
☐	☒	The exact sample size (*n*) for each experimental group/condition, given as a discrete number and unit of measurement
☐	☒	A statement on whether measurements were taken from distinct samples or whether the same sample was measured repeatedly
☒	☐	The statistical test(s) used AND whether they are one- or two-sided<br>*Only common tests should be described solely by name; describe more complex techniques in the Methods section.*
☒	☐	A description of all covariates tested
☒	☐	A description of any assumptions or corrections, such as tests of normality and adjustment for multiple comparisons
☐	☒	A full description of the statistical parameters including central tendency (e.g. means) or other basic estimates (e.g. regression coefficient) AND variation (e.g. standard deviation) or associated estimates of uncertainty (e.g. confidence intervals)
☒	☐	For null hypothesis testing, the test statistic (e.g. *F*, *t*, *r*) with confidence intervals, effect sizes, degrees of freedom and *P* value noted<br>*Give P values as exact values whenever suitable.*
☒	☐	For Bayesian analysis, information on the choice of priors and Markov chain Monte Carlo settings
☒	☐	For hierarchical and complex designs, identification of the appropriate level for tests and full reporting of outcomes
☒	☐	Estimates of effect sizes (e.g. Cohen's *d*, Pearson's *r*), indicating how they were calculated

Our web collection on statistics for biologists contains articles on many of the points above.

Software and code

Policy information about availability of computer code

Data collection	No software was used for data collection.
Data analysis	Data analysis was conducted using MATLAB version R2023b.

For manuscripts utilizing custom algorithms or software that are central to the research but not yet described in published literature, software must be made available to editors and reviewers. We strongly encourage code deposition in a community repository (e.g. GitHub). See the Nature Portfolio guidelines for submitting code & software for further information.

Data

Policy information about availability of data

All manuscripts must include a data availability statement. This statement should provide the following information, where applicable:

- Accession codes, unique identifiers, or web links for publicly available datasets
- A description of any restrictions on data availability
- For clinical datasets or third party data, please ensure that the statement adheres to our policy

All data is available in the main text or the supplementary information.

Research involving human participants, their data, or biological material

Policy information about studies with human participants or human data. See also policy information about sex, gender (identity/presentation), and sexual orientation and race, ethnicity and racism.

Reporting on sex and gender	N/A
Reporting on race, ethnicity, or other socially relevant groupings	N/A
Population characteristics	N/A
Recruitment	N/A
Ethics oversight	N/A

Note that full information on the approval of the study protocol must also be provided in the manuscript.

Field-specific reporting

Please select the one below that is the best fit for your research. If you are not sure, read the appropriate sections before making your selection.

[x] Life sciences [ ] Behavioural & social sciences [ ] Ecological, evolutionary & environmental sciences

For a reference copy of the document with all sections, see nature.com/documents/nr-reporting-summary-flat.pdf

Life sciences study design

All studies must disclose on these points even when the disclosure is negative.

Sample size	Ex-vivo sample size was at least 3, based on previously published literature on similar evaluations. Live animal sample size was limited by animal availability. Squid and jellyfish sample sizes were each greater than 3.
Data exclusions	No data was excluded.
Replication	Rapid sensor adhesion to each marine species was reliably reproduced using the same application method. Each experiment was replicated two to five times on distinct samples of the same group.
Randomization	All tests were performed with randomly allocated experimental groups.
Blinding	Blinding was not done for data directly reported without processing (i.e tag attachment times). Tagged and untagged animals were chosen at random from the tank experiments. For the animal movement analysis, sensor data was identified by the sensor number instead of the species until after data was processed. Additionally, all animals, whether tagged or not tagged, were subject to identical handling procedures (more detail in the methods section).

Reporting for specific materials, systems and methods

We require information from authors about some types of materials, experimental systems and methods used in many studies. Here, indicate whether each material, system or method listed is relevant to your study. If you are not sure if a list item applies to your research, read the appropriate section before selecting a response.

Materials & experimental systems

n/a	Involved in the study	
[x]	[ ]	Antibodies
[x]	[ ]	Eukaryotic cell lines
[x]	[ ]	Palaeontology and archaeology
[ ]	[x]	Animals and other organisms
[x]	[ ]	Clinical data
[x]	[ ]	Dual use research of concern
[x]	[ ]	Plants

Methods

n/a	Involved in the study	
[x]	[ ]	ChIP-seq
[x]	[ ]	Flow cytometry
[x]	[ ]	MRI-based neuroimaging

Animals and other research organisms

Policy information about studies involving animals; ARRIVE guidelines recommended for reporting animal research, and Sex and Gender in Research

Laboratory animals	This study did not involve laboratory animals.
Wild animals	Squid, skate, sea bass, sea robin and flounder were obtained from the Marine Resources Center at the Marine Biological Laboratory, Woods Hole, MA. Jellyfish (moon jellyfish, 6" bell diameter) were obtained from Sunset Marine Labs, transported overnight in water bags and acclimated in saltwater tanks before testing. Lobster was purchased from the local fish market. Skate, flounder, sea robin and sea bass were released into the nearby vineyard sound two weeks after testing. Local squid, jellyfish, and lobster were kept in their tanks indefinitely after testing. Squid in the Azores were caught by jigging and released upon tag application. Skate, squid, flounder, and salmon tissues for ex-vivo testing was purchased from the local fish market. Animals for live testing were wild-caught, although all animals were estimated to be fully grown adults based on size.
Reporting on sex	Gender was not considered in this study as a variable that would affect BIMS performance due to similarities in topological features across genders for the species tested. Additionally, most species were not obviously sexually dimorphic. Squid for example do not display sex organs during the time of experiment. Thus, identifying individual sex would require invasive probing and prolong tagging processes, which opposed a key goal of BIMS to rapidly and non-invasively adhere sensors.
Field-collected samples	All live animals used for tank testing were kept in UV filtered seawater tanks at 18 C and allowed to acclimate for at least 24 hours prior to any testing. Wild caught squid in the Azores was caught via jigging, tagged and immediately released.
Ethics oversight	This study was carried out in accordance with the principles of the Basel Declaration and recommendations and approval of the Woods Hole Oceanographic Institution's (WHOI's) Institutional Animal Care and Use Committee scientific protocol to TAM. IACUC number: 20811.03. Field testing in the Azores was completed with Dr. Pedro Afonso and Dr. Jorge Fontes under the Azorean Regional Government's International Recognized Compliance Certificate 46/2021/DRCTD for access and use of natural resources for scientific purposes.

Note that full information on the approval of the study protocol must also be provided in the manuscript.
